# Designing collaborations involving health technology assessment: discussions and recommendations from the 2024 health technology assessment international global policy forum

**DOI:** 10.1017/S0266462324000436

**Published:** 2024-11-04

**Authors:** Rebecca Trowman, Antonio Migliore, Daniel A. Ollendorf

**Affiliations:** 1 Health Technology Assessment International (HTAi), Edmonton, AL, Canada; 2 Institute for Clinical and Economic Review, Boston, MA, USA; 3 Tufts Medical Center, Boston, MA, USA

**Keywords:** Collaboration, HTA, multistakeholder, international

## Abstract

Although collaboration is an intensive way of working together, it is essential for such efforts to achieve shared goals. Health technology assessment (HTA) is transdisciplinary and has an important history of collaboration, with collaboration featuring increasingly in the strategic plans of HTA bodies and stakeholders. Collaboration can be between HTA bodies and between HTA bodies and other stakeholders—most notably regulators but increasingly payers, patient and caregiver organizations, clinicians–clinical societies, and academia. The 2024 HTAi Global Policy Forum (GPF) discussed collaborations involving HTA bodies, reviewing existing and previous collaborations to see what has worked and what can be learned. Core discussion themes included: (i) determining the collaboration purpose is essential but may be dynamic, changing over time; (ii) choosing the collaboration topic takes time, requiring upfront investment and stakeholder mapping; (iii) inviting the right participants and treating them equally is important, including those who can impact HTA, those who will be impacted by HTA and those who bring new information; (iv) collaborations need clear governance, defined roles, responsibilities, metrics, and case study–pilots can be a useful operational model; (v) resourcing collaborations sustainably is a challenge—the time, people, and money required are often under-estimated; (vi) undertaking continual, iterative learning reviews ensures ongoing value and impact of collaborations. Recommendations for future work include the development of a “go/no-go” checklist to determine when collaboration is needed, supplemented with a set of “best practice” principles for establishing and working in collaborations involving HTA bodies.

## Introduction

The Oxford English dictionary defines collaboration as the “act of working with another person or group of people to create or produce something,” with everyone working together towards a shared goal (1). Collaboration is now considered an accepted practice within all fields of science. While collaboration is typically considered to be the more intensive form of working together, in contrast to coordination and cooperation (2), given the increasing interest and potential value of collaborating, it was the focus of the 2024 HTAi Global Policy Forum (GPF) (3).

HTA is a “multi-disciplinary process that uses explicit methods to determine the value of health technology at different points in its lifecycle. The purpose is to inform decision-making to promote an equitable, efficient, and high-quality health system.” (4) HTA therefore has a generally consistent aim, globally, to provide fair, equitable access to safe and cost-effective technologies for patients and is by nature transdisciplinary, which necessitates collaboration (5). The field of HTA, therefore, has a long history of collaboration, and participation in collaborations increasingly features in the strategic plans of HTA bodies and HTA stakeholders. Collaboration has taken many forms, including between HTA bodies within and across countries and between HTA bodies and other stakeholders most notably regulatory authorities (6), but increasingly others such as payers, patient and caregiver organizations, clinical societies, and academia.

As noted in an article by Bump et al. in 2021 (7), the drivers of collaboration have remained largely unchanged since the conceptualization of the term in the 19th century. Many of these drivers apply to the field of HTA; according to a European Network for HTA (EUNetHTA) white paper (8), drivers of collaboration within HTA include:

- increased efficiency and quality;
- shared knowledge with expertise and skills leveraged across organizations;
- increased credibility for the individual HTA body;
- improved timeliness with collective, rather than individual, effort;

Most examples of collaboration in HTA to date have related to sharing knowledge, experience, and comparing information and processes. “Designing the rhythm” for successful collaborations involving HTA bodies was chosen as the 2024 GPF topic because of the volume of existing and new collaborations and the desire by GPF members to review what has worked well and what learnings can be applied to future efforts. Many multi-stakeholder collaborations often fail to transform the “rhetoric into reality” (9) and collaboration is felt by some to have become so pervasive that the term is now used liberally referring more generally to any form of working together (10), (11). Further, collaboration within HTA bodies and other organizations within the HTA ecosystem can be limited; for example, with separated teams working on planning, appraisal of clinical effectiveness, and cost-effectiveness, with teams often working across different technology types and stages. The goal of the GPF discussion was to identify the factors that are associated with meaningful collaborations, to enable future collaborations to be set up for success from inception.

The GPF Background Paper (12) collated information available in the published literature obtained using a targeted literature review. This was supplemented by semistructured expert interviews and included details of examples of collaboration involving HTA bodies. The interviews were conducted with identified and nominated experts in the field, many of whom were involved in multiple collaborations and spoke from various perspectives. To guide the interviews, a desktop review of collaborations that each individual was involved with was conducted and the interviews were then guided by a typology that essentially focussed on the “who, what, and how” of each collaboration, and interviewees were asked to describe these elements of their relevant collaborations plus a description of barriers and enablers.

This typology was then further developed in the background paper to enable the GPF to explore how current collaborations have been constructed and what their key features are, what has worked, what continues to work (i.e., what are the drivers and conditions according to each typology), and what might need to change. The focus was on determining the types of activities (corresponding to the lifecycle of a technology) that may be best suited to multi-stakeholder collaborative efforts and, if possible, to develop conditions that could be applied according to the typology to ensure that future collaborations are successful.

## GPF meeting structure

Over twenty-seven to twenty-nine January 2024, eighty representatives from not-for-profit organizations (HTA agencies, payers, and health systems), for-profit organizations (pharmaceutical, biotech, and device companies), patient representatives, invited speakers and HTAi leadership met in San Diego, USA for the 21st annual HTAi GPF. The meeting was conducted under the Chatham House Rule (13), whereby participants are free to share information obtained at the meeting, but they may not reveal the identity or affiliation of the person providing the information. This paper presents the authors’ view on the 2024 GPF and is not a consensus or official statement from individuals who attended the meeting or their organizations.

The GPF began with a keynote and a spotlight presentation highlighting the work of two collaborative efforts that are both global in reach, well-established, and relate (and to varying degrees) include HTA bodies and the HTA community. The first was the NEW Drug Development ParadIGmS (NEWDIGS) (14) initiative that aims to improve health outcomes by accelerating appropriate and timely access for patients to biomedical products and developing innovative solutions to problems that are too complex and cross-cutting to be addressed by a single organization or market sector. The second was from the clinical trials transformation initiative (CTTI) (15), a multi-stakeholder forum that aims to develop and drive the adoption of practices that will increase the quality and efficiency of clinical trials.

These presentations highlighted the importance of taking time to select topics for collaboration and how to ensure the right people are involved. Furthermore, the concept of looking at whether more collaboration could happen “upstream” of traditional HTA with projects focused on the readiness of the market for new technologies (as opposed to the readiness of technology for the market) was discussed. These presentations were followed by a multi-stakeholder case study and panel session that included patient, HTA body, and industry perspectives on relevant and recent examples of collaboration—including the European HTA Regulation (16) and the collaboration of nonEuropean HTA bodies (informally known as the AUS-CAN-NZ-UK collaboration) (17).

Following moderated plenary discussions, the GPF members were divided into six breakout groups to discuss challenges and opportunities related to the topic themes and develop priority recommendations for action. The themes provided to the breakout groups are described in Table 1.

**Table 1.** Breakout discussion themes and prompts

Groups	Prompts session 1	Prompts session 2
Group 1 – Collaborations between HTA bodies (national, regional, and international) Group 2 - Collaborations involving HTA bodies and organizations that can directly impact HTA systems (i.e., regulators, payers, academia, policy-makers) Group 3 – Collaborations involving HTA bodies and organizations that are impacted by HTA systems (i.e., patient organizations, industry, guideline developers, clinicians, health systems – these are typically larger multi-stakeholder collaborations)	Discuss the overall aims, goals, and priorities for the type of collaboration that you are focusing on. In other words, why do it? What is the aim of this type of collaboration, and what do different stakeholders expect regarding their involvement? How can the performance, value, and impact of collaboration in HTA be measured? Is there a natural or even hypothetical counterfactual that could be considered? How should we define ‘good’ collaboration of this type? What are some of the ‘best’ examples of collaborations of this type that have worked well? Why do these collaborations fail? Are there elements of current collaborations within HTA that can be improved upon? Are there different aims and objectives according to the topic of collaboration? Are there situations/activities that are particularly suited to collaboration? Are there types of stakeholders for each collaboration format who should routinely be included? Not explicitly included but informed? Excluded?	Part 1: Can the aim of the type of collaboration (discussed yesterday) be defined concisely? (i.e., What is the “elevator pitch” for this type of collaboration)? Part 2: Are there any good practice guidelines or principles that could be produced, according to the type of collaboration discussed in your group? Consider: Structure of governance and day-to-day leadership Optimal duration Description of roles and responsibilities Ensuring accountability Outputs and dissemination
Group 4 – Situations of where collaboration is not, or no longer, needed or likely to add value	Identify specific areas where it seems that [further] collaboration is not necessary or will not add value. Is there ever “too much” collaboration? How do we know when to stop collaborating? Considering cooperation and coordination as “lower intensity” activities; are there particular activities that are better suited to these ways of working together? What are they? Should smaller collaborations be streamlined and convened in a more systematic way? How do we avoid duplication of collaborative efforts (that is multiple collaborations working on the same or similar topic)? Are there ways of working together that might be appropriate for different lifecycle activities and/or according to technology type (e.g., drugs, devices, digital health, etc.,)? Are there some activities that consistently make for an individual HTA body to take on alone?	We have identified situations where collaboration may not be needed (either at all, or may not need to continue)—if some way of working together is still desired, what other steps can be taken to achieve this?
Group 5 – Resourcing collaborations sustainably	What are the key resources necessary to make collaborations work and to sustain them? What resources are minimally required to initiate and then sustain a collaboration (considering jurisdictional contexts, organizational roles and remits, and budgetary constraints) What are the most important incentivization considerations for collaboration when it is not mandated? Why can the process be slow; are there any approaches that could make collaboration more efficient? How can (perceived) conflicts of interest based on funding sources be managed? How can the “return on investment” of participating in collaborations be demonstrated? How could success from one collaboration be transferred to another? How can it/should it be “scaled up” and/or translated into other settings/countries? What are the opportunities for existing collaborations to invite new collaborators (e.g., regulatory collaborations adding HTA and patient participants); what are the conditions and prerequisites for this to be successful?	Part 1: How can the resources to initiate and sustain collaborations be obtained; what are the key efficiency considerations? Do these vary according to jurisdiction/remit/type of collaboration? Part 2: Are there any good practice guidelines or principles for sustainably resourcing collaborations that could be considered (for example, minimum level of investment and personnel, frequency of interaction, division of labor, and rules of engagement)?
Group 6 – Opportunities for, and starting, new collaborations	Identify opportunities for collaboration that have not yet been explored within the HTA ecosystem. Are there any collaborations (within and beyond HTA) that still need to be explored? If so, what are they and what barriers might there be to their creation? Is greater collaboration becoming required with the changing healthcare landscape and development of complex technologies? What is the impact of the COVID–19 pandemic on future collaborations? How can any negative impacts be mitigated? Are there types of stakeholders for each collaboration format who should routinely be included? Not explicitly included but informed? Excluded?	Part 1: Identify priority/key areas for new collaborations involving HTA bodies. Part 2: Are there any good practice guidelines that could be developed for consideration when establishing new collaborations (for example purpose of collaboration, stakeholder mapping, governance, communication strategies, evaluation, and review).

## Meeting discussions

The discussions from the 2024 GPF were wide-ranging, reflecting the multiple types of collaboration included. Details of the discussion by the breakout group are provided below.

### Group 1: Collaborations involving HTA bodies (national, regional, and international)

While the group did not reach a consensus on the specific aims and expectations of collaboration between HTA bodies, some common themes were identified. This type of collaboration was felt to potentially achieve:

- Efficiency gains by sharing work;
- Enable joint methods development and alignment;
- Alignment (and possibly consistency) across HTA bodies on principles for patient involvement, value perspectives, and evidence requirements;
- Enhancement of the purpose of HTA, with closer working with government departments and increased predictability for all stakeholders.

Group one considered that collaboration across HTA bodies could “increase the credibility and quality of HTA services and findings” and that upfront investment to prepare the “why,” the “what,” the “who,” the “how” and the duration of the collaboration is needed. The group noted that given that there is always a trade-off when participating in activities (e.g., from a bandwidth perspective), there is a danger in over-committing, and careful and upfront preparation can be a valuable investment for HTA bodies. Examples of the group discussed included collaborations that had a lack of a clear goal and purpose; in cases like this, the collaborative activities were underway before the participants of the collaboration had really discussed what the end product would be.

Group one highlighted that for collaboration between HTA bodies to be successful, clear governance is essential so that there is clarity on the purpose of the collaboration and clear roles, responsibilities, and leadership. Following this, there should be learning in an iterative way with impact measured regarding the purpose of the collaboration and regular reviews to determine whether to continue, adapt, or stop.

### Group 2: Collaborations involving HTA bodies and organizations that may directly impact HTA (e.g., regulators, payers, and academia)

Group two identified that HTA bodies should collaborate more deliberately and systematically with non-HTA bodies to share respective expertise in the process and improve patient outcomes at a price that systems can afford. However, collaboration does not necessarily mean consensus and different stakeholders will have different perspectives—this should not preclude collaboration.

Furthermore, this group highlighted that open and pragmatic dialogue around the overall goal of the collaboration is needed and incorporating learnings from existing or previous collaborations is important. Measuring the performance of collaborations is difficult, but HTA practitioners are experts in “measuring the unmeasurable” and so the group felt that the HTA community should be able to find ways to do this.

Group two developed five principles (the “5 Ds”) when considering collaborations with HTA bodies and other organizations:

- **Demonstrate** understanding and respect for the roles and remits of each organization;
- **Define** clear objectives and responsibilities on specific topics in order to build trust across the collaboration;
- **Design,** establish, and recruit to roles that enhance the operations of collaborations, including project management, financial, and operational roles;
- **Deliver** and manage behaviors that encourage and facilitate information generation and sharing across organizations; and
- It **depends** on when to collaborate—collaboration may be harder but can be better than “going it alone”; however, it may not always be the best option for working together or achieving a particular outcome.

### Group 3: Collaborations involving HTA bodies and organizations that HTA may impact directly (e.g., patient groups, clinicians, and health systems)

Group three first noted heterogeneity across health systems and structures, and the variability in the maturity of HTA bodies and that this has a direct impact on the available resources to use as inputs in collaboration and the desired outputs. Group three also highlighted that where implementation of any recommendations created through collaboration is voluntary, then who has responsibility for adoption needs to be discussed and clarified at the start of the collaboration. The potential impact of collaborations should be clear and goals should be measurable (e.g., impact on speed, evidence generation, equity of access) with identified mechanisms to translate global thinking into local action.

Group three also felt that stakeholder mapping to identify who to involve in collaboration is a valuable activity and highlighted the following areas of consideration around stakeholder involvement:

- Industry partners can bring in-depth technical knowledge and external perspectives across global HTA bodies.
- Patients are a critical stakeholder group, and continued conversations and recognition of patient contributions are essential. Participatory and codesign models with patients as coleaders are increasing; however, caution around conflicts of interest among some patient groups was noted.
- Equity of access for all stakeholders to participate in collaborations is always needed; this may include logistical adaptations and flexibility where necessary (e.g., language translation, use of sign language, accessible facilities for in-person meetings, and so on).
- There are too few active collaborations including the “end-users” of HTA recommendations such as payers and clinicians.
- The inclusion of multiple participants must be balanced with the size of the collaboration to ensure groups do not become unmanageable in size and/or have a large proportion of observers instead of active contributors. Maintaining local engagement in larger collaborations can be challenging, and feedback loops can help achieve this.
- To maintain the balance of power a neutral party may be required to facilitate collaborative discussions and technical concepts must be well defined to increase inclusivity for all.

### Group 4: Areas where collaboration is not, or no longer, needed

Group four considered when collaboration may not be the best model for working together, and where it is not, felt that this is often due to political or logistical reasons. For example, there are times when trust may be too difficult to achieve, opinions within stakeholder groups may be too entrenched to change practice, the topic requires decisions to be made at a local level, or there may just not be enough time, resources, or expertise to collaborate effectively. In these cases, cooperation, coordination, or even improved communication may provide more appropriate ways of working together.

A core checklist could be developed when considering whether collaboration could be useful. Such a checklist could include elements such as:

- A clear mandate, goal, and purpose for the collaboration that avoids repeating the work of other collaborations;
- Relevance of the topic of collaboration to the stakeholders involved;
- Mutual trust and respect between partners involved in the collaboration;
- Sufficient numbers and diversity of stakeholders to make the collaboration credible;
- Having outcomes that are realistic and implementable.

Replicating the efforts of collaboration—noted as distinct from repetition—can be valuable if there are new lessons and learnings to be gathered. This can be achieved by conducting the collaboration again in a different setting, or by adding or developing further elements to be tested.

### Group 5: Resourcing collaborations sustainably

Group five considered the resources required for a collaboration depend on the complexity and duration of the collaboration. Short-term efforts can be easier to create, easier to maintain engagement in, and can be less resource-intensive. Longer-term efforts are harder to sustain given the investment required. Broadly, the group identified three types of resource requirements for collaborations:

1. **People:** having the right people with junior and senior profiles is needed and this is likely to require capacity building. Internships and mentoring programs across organizations could provide one solution but could require confidentiality agreements and potentially have a negative effect on staffing levels. Independent entities, such as the International Network of Agencies for Health Technology Assessment (INAHTA), could assist in information sharing and professional development.
2. **Time:** collaborations take time to become established and to deliver results, but many have an ultimate goal of making processes and systems more efficient and thus saving time. There is a tension between a need for speed and desire to collaborate and this can be compounded by competing interests across, and even within, stakeholder groups.
3. **Funding**: with limited money available to conduct business as usual, participation in collaborations needs to be carefully considered and when embarked upon should be prioritized. The source of funding for collaborations remains an area of debate; for example, should industry funding be handled through a centralized entity to reduce potential conflicts?

As noted by other groups, group five also recommended that the definition of the goal and purpose of a collaboration should always be established upfront and that preinvestment is likely to be needed to determine the need and potential value of collaboration. Resources are also needed for ongoing participation and engagement, with collaborations requiring almost daily nurturing to be successful. All of these factors must also occur in alignment, with all participating organizations being ready to collaborate at the same time as one another. This can be facilitated by ongoing opportunities to network, building trust and relationships across HTA bodies and beyond. Understanding where there are commonalities across HTA bodies, for example in methods and how these have evolved; as recently reviewed by the Office of Health Economics (18) can also help. Dissemination of recommendations also frequently takes more resources than estimated.

### Group 6: Opportunities for new collaborations

Group six focused on how to determine what new collaborations might be needed and felt that to do this HTA bodies first need a process to understand what they are already doing, that is, taking stock of formal and less formal participation in collaborative efforts, and then prioritizing any identified collaboration topics. These processes must include both internal and external perspectives. Group six highlighted that there is a risk that a proliferation of unnecessary collaborations could result in multiple, smaller networks within larger regional or international networks working on very similar topics. This could result in duplication of effort, wasted resources, and potentially staff spread too thinly across efforts.

Group six suggested that the decision to be involved in future collaborations could involve a combination of strategic short and long-term activities, for example, the creation of infrastructure for ongoing multiple collaborative projects or involvement in a one-off collaboration. Furthermore, the topic should be chosen based on an alignment of interests and where building a common approach is likely to be feasible. As mentioned by other groups, group six also recommended that once topics are prioritized, a clear aim and timeline with metrics for success should be defined, stakeholder mapping conducted to identify relevant participants, governance defined, and resources obtained.

Some potential areas that could benefit from further collaboration among the HTA ecosystem identified by group six included:

- Development of scientific guidance on issues such as the use of data for disinvestment decisions with payers;
- n of 1 trials and how regulators and HTA bodies will become ready for these (noting that HTA bodies may not necessarily lead these collaborations but need to be included in the developments);
- Professional development within HTA bodies and other stakeholder groups, for example, creation of training programs and fellowships, use of grassroots efforts and user groups
- Joint HTA outside of the European Union, and where could bring more clarity and less resource wastage during trial design—particularly for industry.
- Future collaborations with providers and payers, for example, around procurement. This could include broadening conversations to disease and treatment pathways instead of discussions around individual molecules/technologies.
- Further collaborations on patient engagement and involvement with the HTA process; may also include furthering methodology around measuring quality of life—particularly in patients who may be less familiar with the HTA process and less willing/able to engage than others.
- System readiness for new technologies; the concept that more collaboration could allow system stakeholders to prepare health systems for innovation using HTA, rather than applying HTA to individual technologies to see if and when they are ready for use in a health system.

## Recommendations and next steps

A summary of the key discussion points emerging from the GPF discussions can be found in Table 2.

**Table 2.** Summary of key breakout group discussion points

Theme	Discussion summary
Why	Determining the purpose of a collaboration is an essential first step; however, is often overlooked. The “why” may be dynamic and change over time and can help determine the level of involvement an organization may have in a collaboration. Examples of why HTA bodies and others collaborate include efficiency gains, reduced duplication, and information sharing, often with the ultimate aim of accelerating patient access to the right technologies in an equitable and sustainable way.
What	Identifying and selecting the topic for a collaboration is essential and takes time, upfront investment with stakeholder/content mapping. Some topics are better suited to other ways of working together such as cooperation or coordination (for example where opinions are too entrenched or the solution is needed too quickly for a collaboration to develop). A list of suggested future topics that could benefit from collaborative efforts is included in the meeting summary at the end of this section.
Who	Stakeholder mapping is needed to ensure that the right participants are invited. In doing so consideration should be given to who may be impacted by the collaboration outcomes; who may impact them; and who may bring otherwise unknown information. Trust and mutual respect are essential and treating all participants as equals is critical (particularly ensuring this happens for patients). Care should be taken to make sure collaborative meetings do not become too large and that the proportion of “observers” does not outweigh “active contributors.” Including those with project management and financial experience is beneficial.
How	Clear governance structures, defined roles and responsibilities, and transparency about these are needed. Do not underestimate the potential impact of cultural differences and language barriers in a collaboration. Case-study and pilot approaches can be useful, particularly when translating global thinking into local action. Resourcing collaborations sustainably is a challenge but the resources required will depend on the complexity and duration of the collaboration. Broadly, time, people, and money are required to undertake any collaboration and the level required is often under-estimated.
Impact	While challenging to measure, clear metrics for success should be established upfront. The potential impact of collaborations should be clear and goals should be feasible, common for all stakeholders, and measurable (e.g., impact on speed, evidence generation, and equity of access). If the implementation of collaborative recommendations is voluntary, then responsibility for adoption should be discussed upfront. Ensuring that there is a continual review of the work of the collaboration (with potential go/no-go decision points if required) and iterative learning is important to ensure the ongoing value and impact of the collaboration.

From the discussions, two key recommendations for the next steps were suggested during the 2024 GPF. While these require development by a multi-stakeholder task force that extends beyond the HTAi GPF membership, they include (but are not limited to) the following ideas and concepts:

1. A “go/no-go” checklist to assist in considering when collaboration is needed. Such a checklist could include determining the relevance of the topic; the feasibility of collaboration; the likely timelines; and stakeholder mapping. Taking stock of any other relevant initiatives and processes for prioritizing participation in the collaboration could be incorporated into such a checklist.
2. “Best practice” principles for establishing and working in collaborations involving HTA bodies. This could include: clearly defining the purpose, aim, and goals of the collaboration; the duration of the collaboration; key elements of governance structures; identification of relevant partners; definition of roles and responsibilities; determination of group norms (if relevant); value and impact metrics; and evaluation methods. Furthermore, the principles could include guidance on:
  - Acknowledging and overcoming cultural and language barriers; are often under-estimated in current cross-country collaborations;
  - Involving the right stakeholders for the collaboration; including patients, caregivers, clinicians, industry, and payers but also technical experts and those with project management skills;
  - Ensuring patients are treated as equal partners and coleaders where possible, with efforts made to be inclusive and accessible;
  - Opportunities for professional development (within and outside of HTA bodies);
  - Resourcing models that reduce perceived or actual conflicts of interest.

HTAi will consider developing these recommendations in a multi-stakeholder setting, for example in collaboration with other professional societies such as the Drug Information Association (DIA) and the International Society for Pharmacoeconomics and Outcomes Research (ISPOR). Involving organizations such as CTTI and NEWDIGS to leverage their expertise and lessons learned could be an efficient approach that could generate further collaborations within the wider health ecosystem.

## Limitations

This article represents a summary of discussions held at the 2024 HTAi GPF. While this Forum represents a broad range of views and perspectives, the membership of the GPF includes perspectives from countries that primarily enjoy established, mature HTA systems. While informants from beyond the GPF membership were approached for input to the Background Paper prior to the meeting, this is a limitation of the discussion summary as these views were not directly present at the GPF discussions. In low- and middle-income countries (LMICs) and where there are nascent HTA bodies, the value of collaboration may arguably be larger but input from these settings was limited. In these settings, there are greater opportunities for information sharing, training and upskilling, and developing HTA capacity.

Furthermore, the GPF primarily comprises HTA body representatives and life science industry organizations. Patient representatives were specifically consulted during the development of the Background Paper and were invited to the meeting; patient attendees were active participants in the discussions. However, some key stakeholder groups (such as clinicians, payers, regulators, and decision-and policymakers) are less well-represented in the discussions themselves. Greater effort to establish collaborations with these stakeholders was seen as a priority for the HTA community by the GPF membership.

## Conclusion

HTA bodies are already involved in and continue to initiate, many collaborations with a broad range of system stakeholders. A successful collaboration does not imply consensus; it may be necessary to “disagree agreeably” to maintain forward-moving momentum and find practical solutions that can be implemented by participants. As collaboration is fundamentally about human interaction, bringing together diverse perspectives, and taking time to build mutual respect, understanding, and trust with consistent and open dialogue is needed.

There are, however, risks when collaborating, and identifying and stating the aim of any collaboration needs to be done at the outset. This step is, however often overlooked, with the desire to collaborate outpacing the formal establishment. Careful consideration of key factors when setting up and conducting collaborations is needed to ensure that collaboration—as opposed to coordination, cooperation, or communication—is warranted and represents a valuable investment of scarce resources. Even though all HTA bodies face challenges such as resourcing, timeliness, and conflicts of interest when collaborating, the value of collaboration can be long-term and far-reaching.
